# Bioimpedance sensor to detect water content in milk based on van Der Pauw method

**DOI:** 10.1049/nbt2.12056

**Published:** 2021-05-15

**Authors:** Masoomeh Ashoorirad, Rasool Baghbani, Mohammad Reza Ghalamboran

**Affiliations:** ^1^ Department of biomedical Engineering Hamedan University of Technology Hamedan Iran; ^2^ Department Plant Physiology & Biotechnology Life Sciences and Biotechnology Faculty Shahid Beheshti University SBU Tehran Iran

## Abstract

Milk fraud poses serious problems for the dairy industry and consumers' health. The main aim of this study was to reveal the effect of water added to milk by measuring its electrical impedance spectrum. The required sensor was designed based on the van der Pauw method to measure the electrical conductivity of milk at the frequency band of 10 Hz to 5 MHz. The bioimpedance spectrum of the milk of five different cows showed that the electrical impedance spectrum has a high potential for detecting water added to the milk (P < 0.01). The area under the Nyquist curve was introduced as a suitable index to detect water‐added milk. In addition, the characteristic frequency of the bioimpedance spectrum was used as an important index to differentiate water‐added milk from waterless milk. An electrical model was introduced to interpret the amount of water added to the milk using the characteristic frequency. Results showed that it is possible to detect raw milk from boiled milk by measuring its electrical impedance.

## 1 introduction

Milk is one of the dairy industry products with high production and high consumption. World milk production is approximately 550 to 600 million tonnes. The FAO reported that ‘World dairy trade in 2020 is forecast to grow to 78 million tones (milk equivalent), up 1.5% percent year‐on‐year, nearly equal the average growth rate for the preceding five years’ [1]. Therefore, increasing its production leads to great economic benefits. Excessive milk production and consumption make it very challenging to control and establish safety standards. The most common fraud in milk production is an increase in its volume. Fraudsters often use water to increase the volume of the milk. Another type of fraud in the milk is to add starch, caustic soda, or increase shelf life with substances such as hydrogen peroxide, urea, and formaldehyde order to restore and maintain the physical and chemical properties of milk [2, 3]. The effects of substances added to milk on human health are different for each substance. Sodium hydroxide and hydrogen peroxide cause gastrointestinal irritation, nausea, and vomiting. Formaldehyde increases abdominal pain, allergies, and even the risk of gastrointestinal cancer [4].

Various methods have been used to investigate fraud in milk, including Fourier Transform Infrared (FTIR) spectroscopy [5], high‐performance liquid chromatography (HPLC) [6], capillary electrophoresis [7], laser induced breakdown spectroscopy (LIBS) [8, 9], and freezing point instruments [10]. These strategies produce precise outcomes. However, the large number of samples required for analysis, the use of complex and bulky equipment, the time delay in providing results, and the required expensive consumable materials increase the economic and time costs of analysis.

Electrical impedance spectroscopy is an efficient and cheap method to detect milk adulteration. In general, impedance measurement can be performed in two ways: two‐electrode measurements and four‐electrode measurements. In the two‐electrode configuration, the electrodes used for excitation and recording are identical; therefore, in this method, the measured impedance is affected by the contact impedance between the surface of the electrodes and the measured material. In the four‐electrode measurements, two electrodes are used for excitation, and the other electrodes are used for recording. Thus, the measurement is accompanied by less error because the contact impedance does not affect on the measurements [11, 12, 13].

Many studies have been done to detect milk fraud using impedance‐based methods. In this regard, Durante et al. measured the electrical impedance spectrum of raw calf milk and heated milk samples through additives such as water, hydrogen peroxide, sodium hydroxide, and formaldehyde. They distinguished the purified samples from the modified samples using the KNN classification method [14].

R.Pethig et al. measured the reactance and electrical conductivity of milk and cream samples with a fat content of 0.15% to 51% (w/w) over the frequency band of 5 Hz to 200 kHz. In this study, they presented a more accurate mathematical relationship for the variation of the conductivity in milk and cream as a function of fat‐free content than that described by Prentice [15]. The main limitation of this study is the use of a two electrode method to measure electrical impedance in which the contact impedance of the electrode with milk, or in other words polarization of the electrodes, affects the measurement results.

Gupta et al. proposed a new handheld microelectrode sensor based on unlabelled spectroscopy to detect adulteration in milk. Their results showed that changes in the concentration of starch and detergent in milk samples significantly influenced the impedance measured by the proposed sensor. They proved that the measured values of electrical impedance are inversely proportional to the concentration of detergent and directly proportional to the concentration of starch in milk [16]. The main limitation of this study is the investigation of milk fraud based on direct values of electrical impedance instead of electrical conductivity because the amount of impedance also depends on the sample size and geometric factor and therefore, the results will not be accurate.

Chakraborty et al. claimed to have built a low‐cost and portable instrument by using the measured impedance phase capable of detecting five types of adulterants (NaOH, NaHCO_3_, (NH4)_2_SO_4_, and NaCl) in milk with different fat content (1.5% to 20%) [17, 18, 19]. The impedance sensor used was a hardwood rod with two back‐to‐back copper electrodes coated on epoxy. This sensor consists of two electrodes for measuring impedance, and therefore, the measurements accompany by an error resulted from contact impedance and electrode polarization.

The Wiener array is the most popular shape of the four‐electrode configuration, in which four electrodes are placed on a straight line with a known distance from each other. Determining electrical properties by Wiener's array requires measured medium dimensions that are quite large relative to interelectrode spacing; if this condition is not satisfied, the measured electrical properties accompany by a large error [20]. Thus, a proper method is needed for measuring the electrical properties of the materials with small volume like milk.

The van der Pauw (VDP) method is a four‐electrode arrangement in which four electrodes are embedded borders of a measured sample. This technique may be used for small volume samples because the measuring electrodes surround the measured medium. Van derPauw presented a worth mathematical proof introducing a technique to measure resistivity and Hall coefficient in small‐thickness semiconductor samples with arbitrary form [21, 22, 23], [24], [25].

Moron showed that the van der Pauw's technique could measure the electrical resistivity of electrolyte solutions and applied this method to determine the electrical conductivity of a salt solution. [21, 26, 27].

Zhang et al in [28] introduced a system for measuring electrolyte conductivity according to van der Pauw's theory. In this research, temperature compensation and its influence on the conductivity measurement and measurement repeatability were studied.

Milk is an emulsified colloid of liquid butterfat globules dispersed within a water‐based solution. Although milk is not homogeneous in terms of its components, measuring the electrical resistivity of milk determines an effective or apparent real value that can be used to evaluate it.

Here, the electrical properties of milk are measured using the VDP method. The electrical impedance spectrum of the samples of cow's milk in different statuses was measured using the proposed VDP sensor. The measured impedance spectrum investigated the effect of adding deionized water to milk. In addition, the electrical impedance of the raw and boiled milk was studied. Finally, an electrical model was introduced; the effect of the adding of the different amounts of deionised water to milk on the measured impedance was investigated using the characteristic frequency and area under Nyquist curve (AUNC) indexes.

## 2 MATERIALS AND METHODS

### 2.1 Theoretical aspects

In this paper, a VDP bioimpedance sensor was constructed to measure the electrical impedance of milk to determine its electrical properties.

**FIGURE 1:**
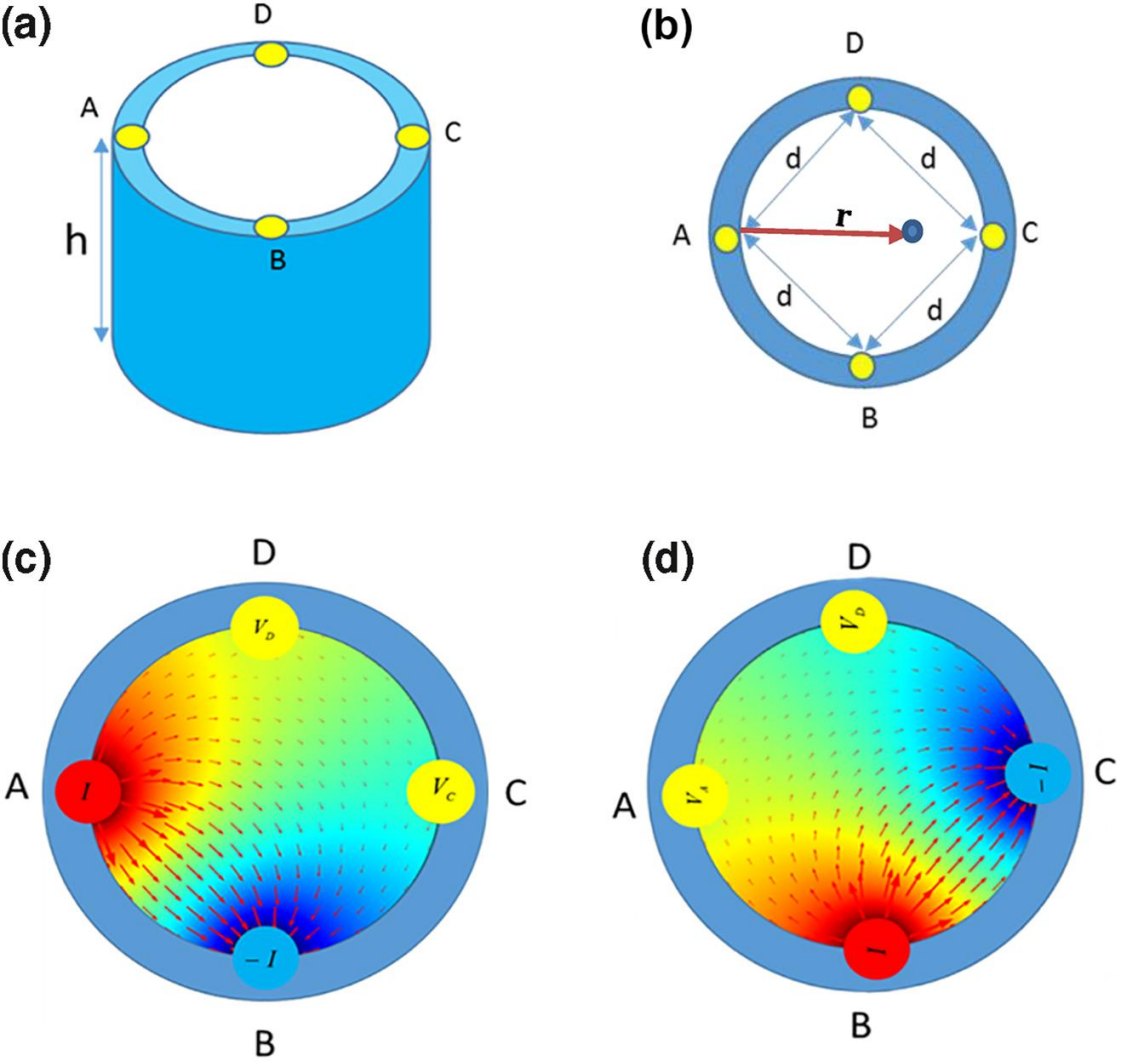
(a) The structure of VDP sensor, (b) Cross‐section of VDP chamber and electrodes (c) Electric field distribution in the first measurement (d) Electric field distribution in the second measurement

In the proposed VDP method, all four electrodes place in the premium of a solid or liquid environment; two electrodes are used to inject current, and the other two electrodes are used to read the voltage (Figure 1(a),(b)).

In Figure 1(c), first, it is assumed that the current exists from electrode A and enters electrode B. To determine the potential difference between the two electrodes C and D, $V_{DC}$, it is assumed that half of the electrode surface is in contact with the solution inside the cylinder chamber (the solution has an electrical conductivity of σ and a height of h). In this case, the current density and the electric field at a distance r from the electrode A are determined as follows:

(1) $$J=\frac{I}{\pi rh}$$

(2) $$E=\frac{J}{\sigma }$$

The electrical potential difference $V_{DC}$ resulting from the current I is determined as follows:

(3) $$\begin{array}{l} {\left(V_{D}-V_{C}\right)}_{A}=-\int E.dr=-\int_{C}^{D}\frac{I}{\pi r\sigma h}dr\\ =-\frac{I}{\pi \sigma h}\int_{d\sqrt{2}}^{d}\frac{dr}{r}=\frac{Ln\sqrt{2}}{\pi h\sigma }I \end{array}$$

In addition, the electrical potential difference $V_{DC}$ resulted from the current −I entering to electrode B is determined as follows:

(4) $$\begin{array}{l} {\left(V_{D}-V_{C}\right)}_{B}=-\int E.dr=-\int_{C}^{D}\frac{I}{\pi r\sigma h}dr\\ =-\frac{I}{\pi \sigma h}\int_{d}^{d\sqrt{2}}\frac{dr}{r}=\frac{Ln\sqrt{2}}{\pi h\sigma }I \end{array}$$

Using the superposition theorem, the voltage resulting from the two currents, *I* (exiting from electrode A) and *‐I* (entering electrode B), is added together. Finally, the impedance is determined as follows:

(5) $$Z_{DC,AB}=\frac{Ln2}{\pi \sigma h}$$

In the next step, the current Iis injected into electrode B, and the current −I is released from electrode C. Again, according to the above relationships, the impedance is determined as follows:

(6) $$Z_{AD,BC}=\frac{Ln2}{\pi \sigma h}$$

The relationship obtained for impedance is the same in two steps and we can write:

(7) $$Z_{DC,AB}=Z_{AD,BC}=Z=\frac{Ln2}{\pi \sigma h}$$

(8) $$\sigma =\frac{Ln2}{\pi hZ}$$

(9) $$\rho =\frac{\pi hZ}{Ln2}$$

The Equation (8) and Equation (9) show the respective electrical conductivity and electrical resistivity, which are complex values for biological solutions. This complex number is shown as $\sigma^{*}$, and its value is determined as follows:

(10) $$\sigma^{*}\left(\omega \right)=\sigma +j\omega \varepsilon_{r}\varepsilon_{0}$$

Therefore, by determining $\sigma^{*}$, the electrical properties, that is, the electrical conductivity (σ) and the relative permittivity $\left(\varepsilon_{r}\right)$ of the measured material, is determined.

### 2.2 Impedance spectrum measurement system

**FIGURE 2:**
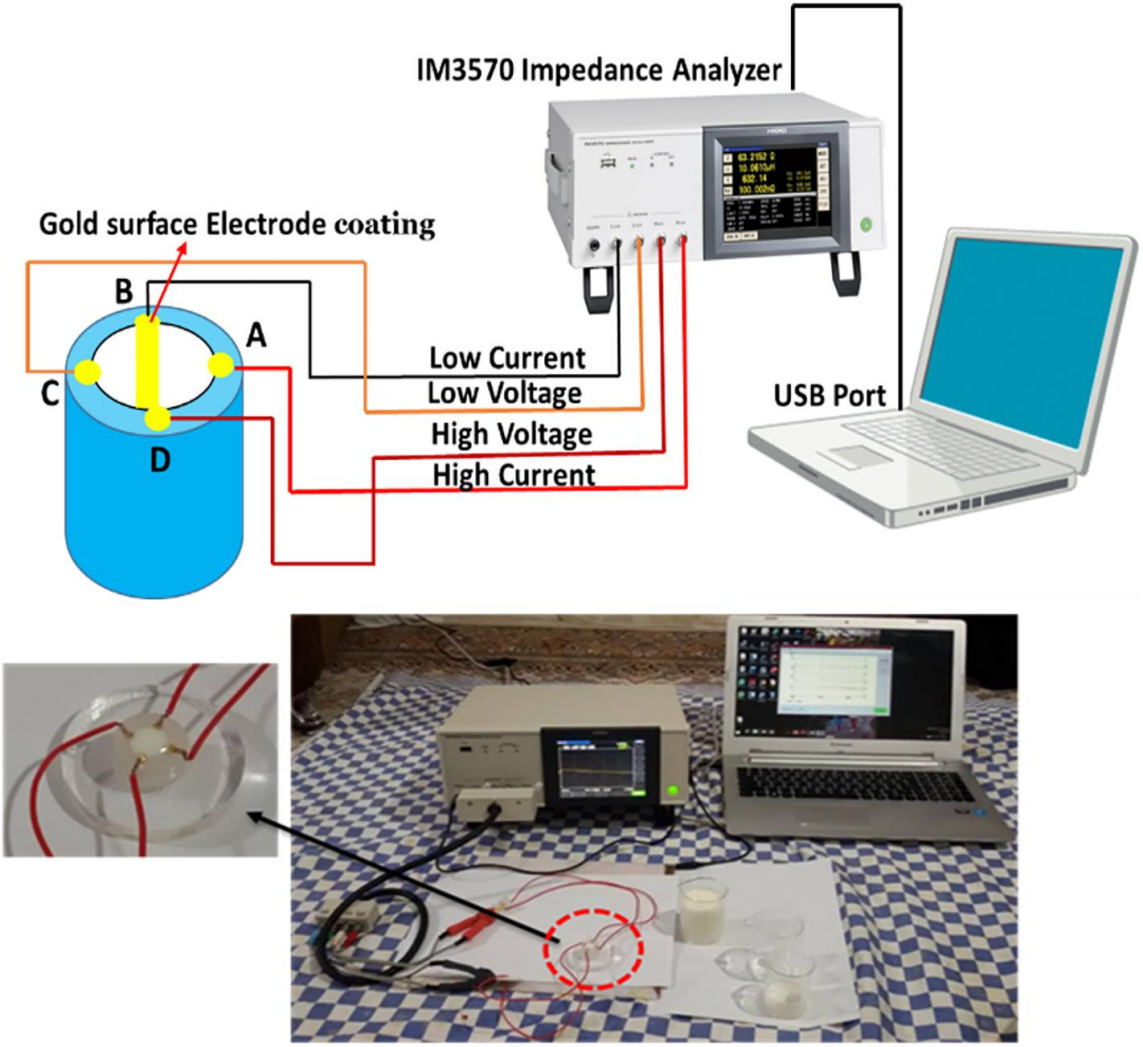
Electrical impedance spectrum measurement setup including three parts: a bioimpedance sensor, an impedance analyser, and a computer or laptop for data acquisition and processing

The measurement system includes three parts: a bioimpedance sensor, an impedance analyser, and a computer or laptop for data acquisition and processing. Figure 2 shows the electrical impedance measurement system for measuring the electrical properties of the milk.

In this system, the VDP sensor had a height and inner diameter of 1 cm; the electrodes' diameter was 3 mm. For the measurement, the four output electrodes were connected to the Hioki IM3570 via four BNC cables, according to Figure 2. Then, the measured values were transferred to the laptop via a USB cable. To reduce the effects of capacitive coupling, the proposed VDP sensor along with the milk sample was placed on the electrical conductor plate connected to the guard port of the IM3570 impedance analyzer. Before the measurement, according to the instructions of the measuring device, the sensor and signal transmission cables were placed in the measurement state; and short‐circuit and open‐circuit calibration was performed to eliminate the noise effects of the cables. Impedance measurements were performed using a sinusoidal stimulation current with a constant amplitude of 1 mA at a frequency range of 10 Hz to 5 MHz at 200 points with linear distances. A rest time of 0.2 s was considered between the two consecutive stimuli. In addition, after each excitation, the measurement was performed with a delay of 0.1s to achieve a stable system state and measurement sample. In addition, to reduce the error, an average of 10 measurements was used.

### 2.3 Preparation of milk samples

Fresh milk samples from five different cows (raw milk) were obtained daily from local animal husbandry. The samples of skimmed milk were prepared by centrifuging the raw milk at 1000 rpm for 3 min. Water‐added milk samples were prepared by adding different amounts of deionized water to raw milk to achieve the required percentages of added water. These samples were carefully stirred to ensure complete mixing and prevent the production of air bubbles, which leads to reduced in conductivity. Immediately after preparing the milk, the temperature was lowered to 4°C (refrigerator). Then, the impedance measurements were performed using the VDP sensor in different modes. Accurate temperature control is essential because it has been shown that the electrical conductivity of milk changes significantly with temperature [25]. Before each measurement, the sensor compartment was washed with diluted detergent, rinsed for at least five minutes with deionized water and finally, dried with hot airflow.

## 3 results and discussion

### 3.1 Impedance measurement of skimmed and raw milk over time

Fat plays a significant role in conduction. The amount of fat in full‐fat milk is about 3.6%, and low‐fat milk is 0.1% [29]. Milk conductivity decreases with increasing fat [10] is because the fat of milk is in the shape of large globules covered by a thin, non‐conductive membranes [29]. These globules fill the volume of the conductive medium and prevent ions from moving between the electrodes. The size of these globules varies from 2 to 10 μm and depends on the cow's breed and the season of the year [10].

**FIGURE 3:**
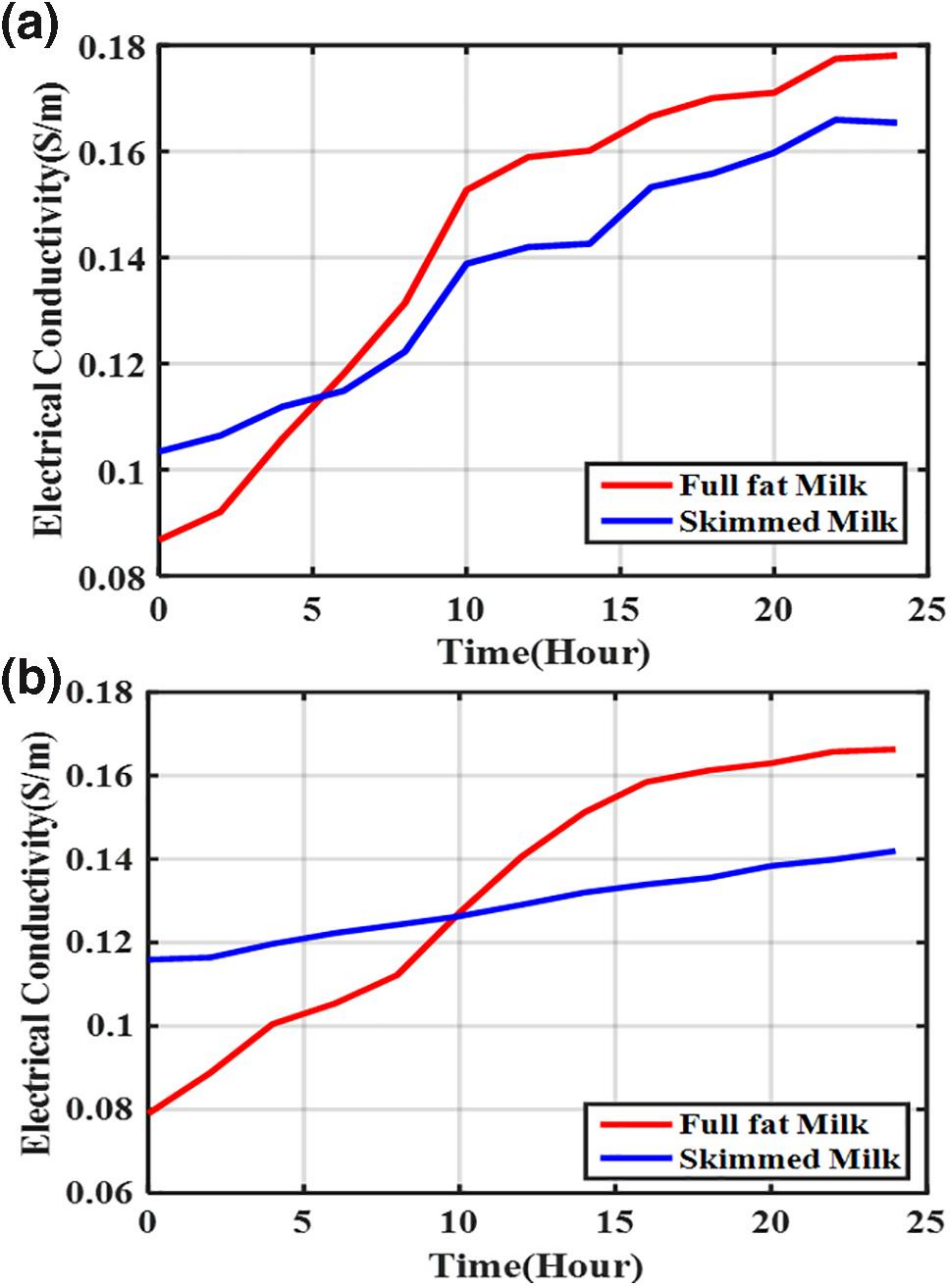
Changes in the conductivity of full‐fat raw and skimmed milk over time for raw milk of two different cows (a) sample 1 (b) sample 2; the milk samples were kept in a refrigerator (4°C) during the measurements

To examine changes in the electrical impedance of cow milk over time, the electrical impedance of five samples of full‐fat raw and skimmed milk was measured at intervals of one hour at a frequency of 100 kHz. Then, the electrical conductivity was calculated according to Equation (8) for all samples of full‐fat raw and skimmed milk. The average of the obtained results for milk samples of two different cows was plotted against time, as illustrated in Figure 3.

As shown in Figure 3, the electrical conductivity of full‐fat and skimmed milk has increased over the first 24 h. The difference in the conductivity of full‐fat milk relative to skimmed milk is due to the accumulation of fat globules, which leads to the breakdown of the thin membrane of fat molecules and almost 50% of the phospholipids in the membrane become free and appear in the form of phosphate ions [29]. Simultaneously, the acidification of milk over time releases calcium ions, and thus, conductivity increases. During acidification of milk, hydrogen ions with a positive charge are released. These ions quickly attach to water molecules and form hydronium ions ($H_{3}O^{+})$ with high conductivity [30]. The effect of fat globules on skimmed milk is negligible because the percentage of its fat is less than 0.1%. As a result, the conductivity of full‐fat raw milk is more elevated than that of the skimmed milk over time. In he first hours, the conductivity of skimmed milk is higher than full‐fat raw milk. This is because the more amount of fat in full‐fat milk has a greater amount of fat than skimmed milk, which decreases the conductivity because the aforementioned phenomena have not yet occurred.

### 3.2 Impedance of boiled and raw milk

**FIGURE 4:**
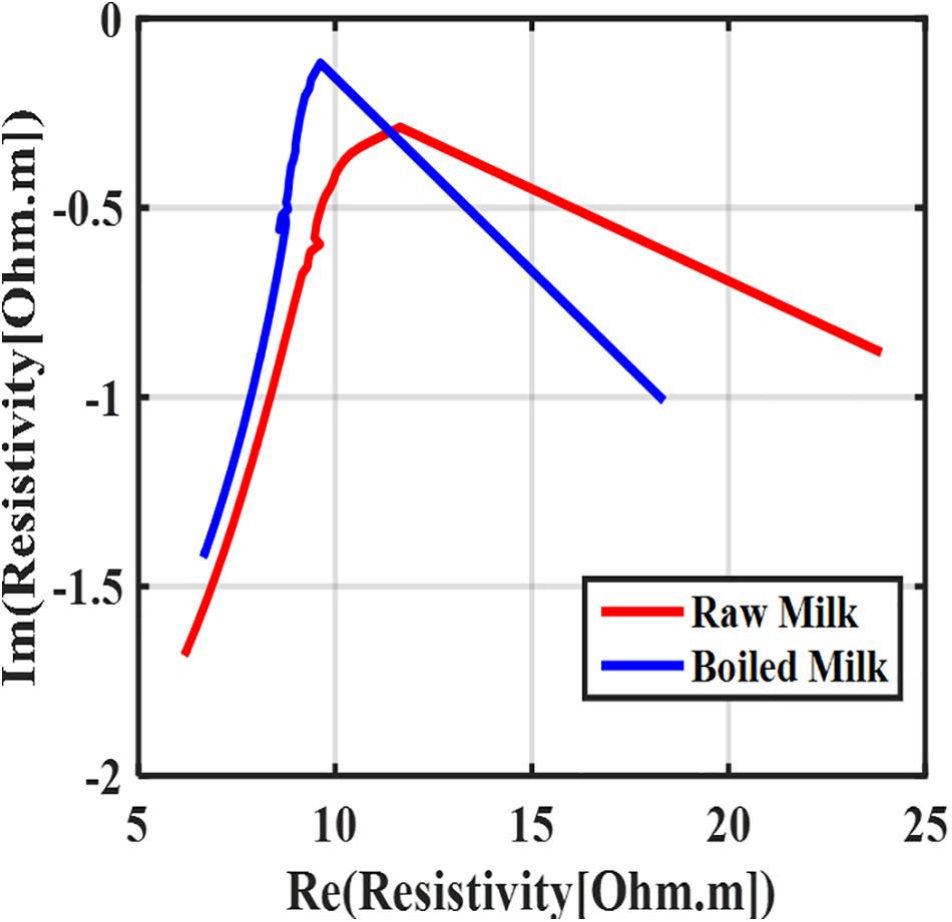
The Nyquist diagram of the measured electrical resistivity of boiled raw milk. The standard deviation from the mean was less than 1%

In this part, a certain volume of fresh raw milk was heated to reach the boiling point. Then the boiled milk was gradually cooled and finally, using a refrigerator, its temperature was reached 4℃ Then, using the proposed measurement system, the electrical impedance spectrum of the samples was measured. The Nyquist diagram of the boiled and raw milk are shown in Figure 4.

As shown in Figure 4, the Nyquist curve of the boiled milk changes in both magnitude and phase concerning to the raw milk. When milk is boiled, its fat content decreases, and as a result, electrical conductivity increases (or electrical resistivity decreased). Fat plays a vital role in conduction. Milk conductivity decreases with fat increasing [3].

For boiled raw milk, the application of heat breaks the bond between the fat molecules, and is broken and the fat mass rises to the milk's surface. Thus, the boiled milk has less non‐conductive fat and more conductivity concerning un‐boiled milk.

### 3.3 Impedance measurement of water‐added milk

The most common substance added to milk is water. Adding water is performed to increase milk volume, which reduces the nutritional value of milk. If the added water is contaminated, it may pose serious health risks. Milk conductivity is mainly determined by charged compounds, such as mineral salts. The salts in milk mainly include chloride, phosphate, citrate, carbonate, and bicarbonate of potassium, sodium, calcium, and magnesium. Although the amount of milk minerals remains constant at about 0.7%, the relative concentrations of different ions can vary and are influenced by animal race, season, food, and stage of lactation [24]. These factors also affect the distribution of calcium, magnesium, and phosphate between the soluble and colloidal phases and thus, the number of free conductive ions in milk.

**FIGURE 5:**
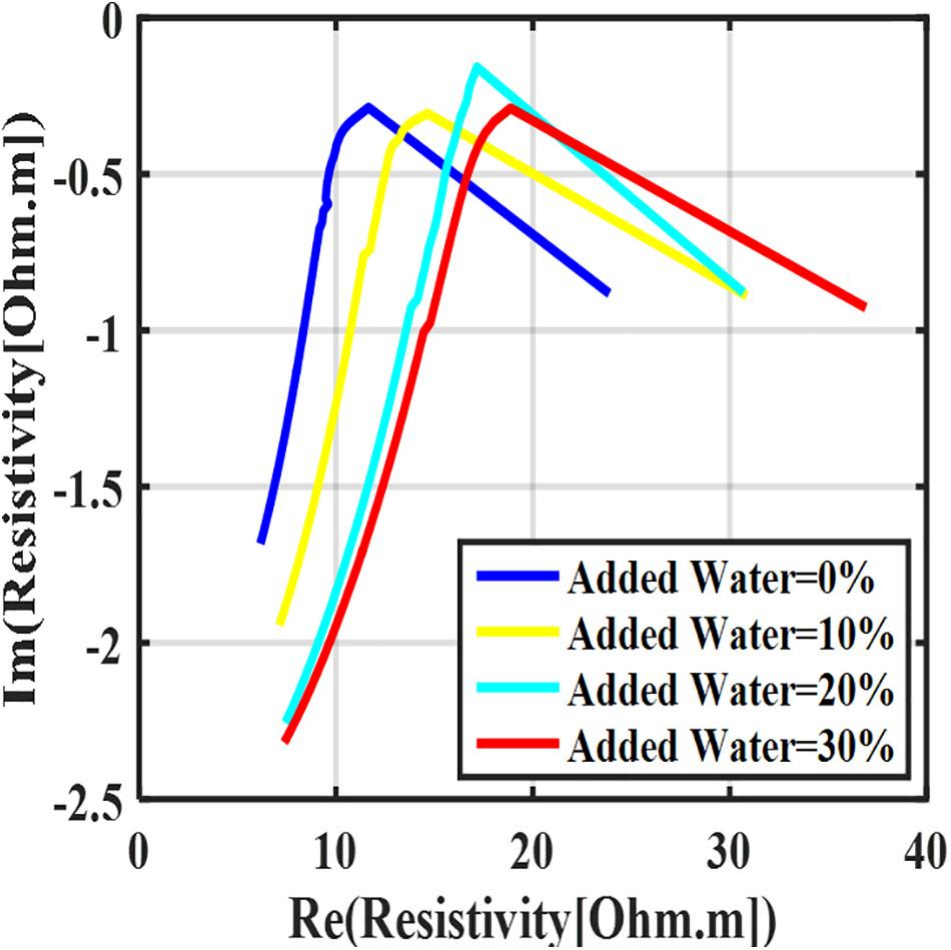
The Nyquist diagram of the diluted raw milk with different percentages of added deionized water. The standard deviation from the mean was less than 1%

When the milk is diluted with water, the concentration of free ions in it decreases, and as a result, its conductivity decreases. In this study, three different concentrations of diluted raw milk were prepared by adding deionized water; then, their electrical impedance spectrum was measured at a constant temperature of 4°C at the frequency range of 10 Hz to 5 MHz. The Nyquist curves of different concentrations of diluted raw milk are shown in Figure 5.

According to Figure 5, by adding deionized water to the raw milk, the Nyquist curve shifts to the right. In other words, with the addition of water, the ohmic resistance of water‐added raw milk increases, and thus, conductivity decreases. By adding deionized water to the milk, the phase of impedance becomes more negative. Water molecules act as dipoles and reveal dielectric properties. The impedance phase becomes more negative when the amount of deionized water in the medium increases [31].

The AUNC in Figure 5 is a proper feature that, which can be used to differentiate the water‐added milk from pure milk. The AUNCs for the five samples of water‐added milk are shown in Table 1. According to Table 1, the AUNC is increased with increases with the percentage of water added to the milk. Thus, the AUNC is a suitable criterion to detect the amount of water added to milk.

**TABLE 1:** Characteristic frequency ($f_{c}\;$) and the AUNC of five different milk samples with different volume percentages of added deionized water

Added Water	Sample 1		Sample 2		Sample 3		Sample 4		Sample 5	
	$f_{c}$	AUNC	$f_{c}$	AUNC	$f_{c}$	AUNC	$f_{c}$	AUNC	$f_{c}$	AUNC
0%	23.25 kHz	11.82	34 kHz	16.56	25.5 kHz	13.57	38.5 kHz	10.29	46 kHz	13.21
10%	22 kHz	17.26	27.75 kHz	19.37	22.5 kHz	17.79	34.5 kHz	15.67	45.5 kHz	16.39
20%	20.7 kHz	19.50	25.5 kHz	27.81	20.25 kHz	19.83	32 kHz	20.51	38.5 kHz	21.48
30%	18.75 kHz	25.86	21.5 kHz	35.92	19.45 kHz	26.43	29.75 kHz	24.97	34.5 kHz	27.28

Abbreviation: AUNC, area under the Nyquist curve.

**FIGURE 6:**
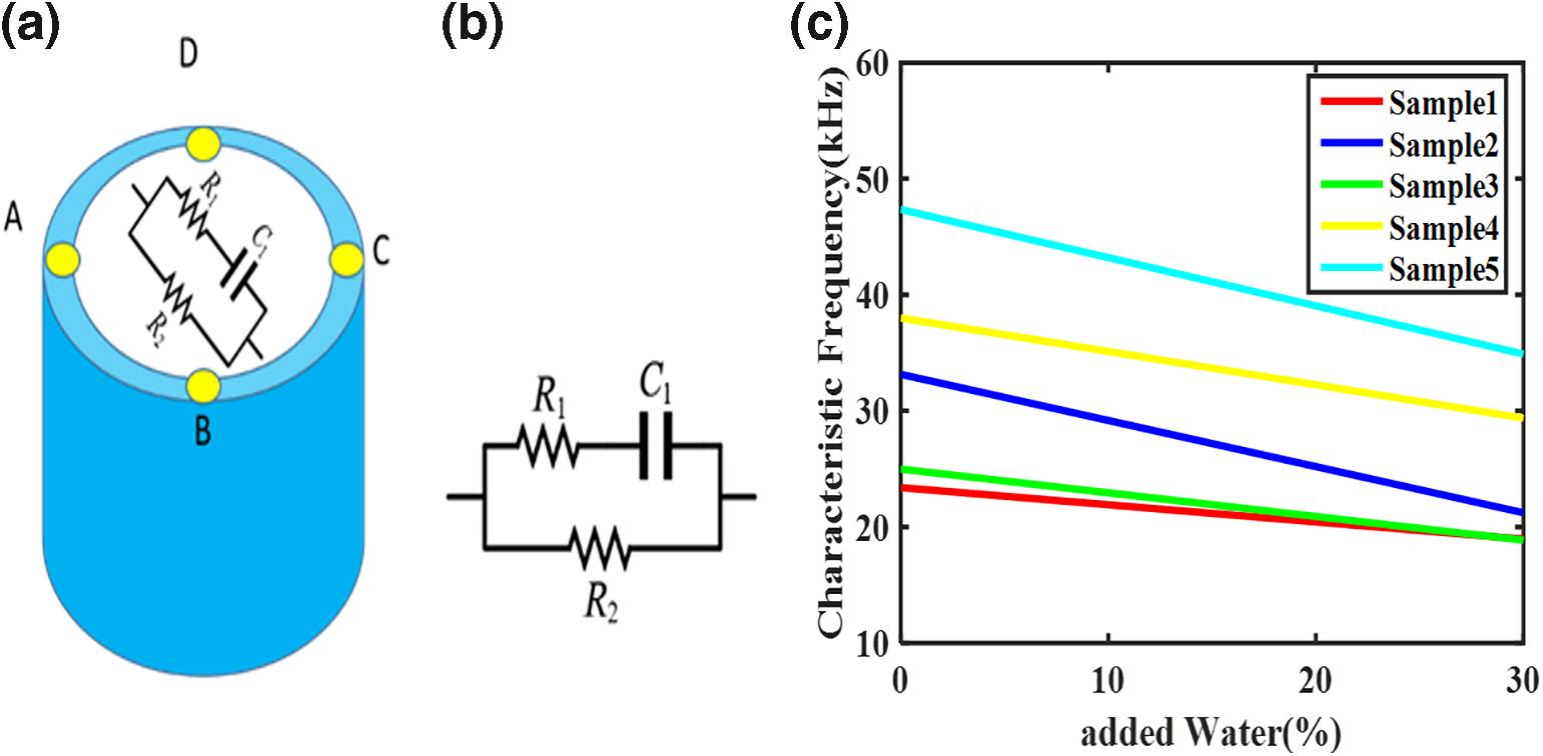
(a) The van der Pauw sensor contains a milk sample (b) Electrical model of milk sample inside the sensor (c) Linear curve fit of the characteristic frequency of the raw milk (4℃) versus the percentage of the added water for five different milk samples with a correlation coefficient ($R^{2}$) of 0.99

For the first time, characteristic frequency is used to reveal the amount of deionised water added to raw milk. Characteristic frequency is the frequency at which the Nyquist curve of the measured impedance of milk samples using the VDP sensor reaches its maximum value. As mentioned in the previous sections, milk consists of fat molecules with thin insulating layers connected to each other (fat globules) and are suspended inside a solution containing various ions. These fat molecules can be modelled with a capacitor $C_{1}$ series with an ohmic resistance $R_{1}$. In addition, the environment around the fat masses contains different ions and is modelled with an ohmic resistance $R_{2}$. Therefore, a simple model of the milk sample inside the chamber of the VDP sensor (Figure 6(a)) can be represented as the electrical circuit of Figure 6(b).

Using the proposed electrical model of the raw milk presented in Figure 6(b), it is easy to determine the characteristic frequency using Equation (11).

(11) $$f_{c}=\frac{1}{2\pi C_{1}\left(R_{1}+R_{2}\right)}$$

According to Equation (11), the characteristic frequency ($f_{c}$) varies by changing the resistances $R_{1}$, $R_{2}$, and capacitor $C_{1}$. By adding the deionized water to milk, the resistance $R_{2}$ increases (ion concentration is decreased), and the resistance $R_{1}\;$ and the capacitor $C_{1}$ remain constant because the amount of milk fat does not change. Thus, the characteristic frequency of the water‐added milk was expected to decrease by adding more water. The measured characteristic frequency for the five samples of water‐added milk with different percentages of water was determined according to Table 1.

Based on the values ​​obtained for the characteristic frequency shown in Table 1, it is clear that the characteristic frequency decreases with the addition of more water to the raw milk. Therefore, the characteristic frequency of milk samples can be used as a proper index for evaluating milk purity. Changes in the characteristic frequency of the raw milk (4℃) in terms of the percentage of the added water for five different milk samples are shown in Figure 6(c). Figure 6(c) shows that by adding water to five different samples of pure raw milk from five different cows of the pure raw milk, the characteristic frequency decreases linearly with increasing water percentage in all five samples. Therefore, the percentage of water added to the milk can be detected by determining the characteristic frequency. According to the characteristic frequencies obtained for five milk samples, the characteristic frequency of raw milk is in the range of several tens of kHz. Thus, the characteristic frequency can be determined with a simple, low‐cost, portable chip such as AD5933, which can measure the electrical impedance spectrum at the frequencies below 100 kHz. Although it is possible to detect the amount of water added to milk by measuring the electrical impedance spectrum of milk, milk's electrical properties depend on many parameters. Some of these parameters can be controlled, such as temperature, while others, such as the amount of fat (which depends on cow's breed, a cow's food, the season, and the stage of lactation), cannot be controlled during measurement. To overcome these limitations, it is recommended that the milk impedance spectrum be measured at its production site at a given temperature.

## 4 conclusion

Based on the results obtained in this study, it was possible to identify milk impurities. With increasing amounts of deionized water, the Nyquist curve of the bioimpedance spectrum shifted to the right, indicating a decrease in conductivity. In addition, the impedance phase was more negative with the addition of more water, resulting in more water dipoles in the environment. The characteristic frequency and AUNCwere used as suitable indicators to detect the amount of water added to milk. By adding more water to the milk, the characteristic frequency decreases and the AUNC increases. The findings of this study can provide an electrical model for interpreting the amount of water added to milk based on the characteristic frequency. Raw and boiled milk can be detected by measuring their impedance spectrum; the boiled milk conductivity rate increased with respect to raw milk due to chemical and physical changes in milk during the heating. In addition, the conductivity of full‐fat and skimmed milk changes over time. The results showed that the impedance spectrum of the milk is a proper technique to detect changes in milk, and thus, it can be used as a low‐cost, effective, and available technique to control milk quality in the dairy industry.
